# Towards Haemoglobin Detection in Finger-Prick Sampling via Low-Cost Disposable Sensor Chips Based on eMIPs on Plasmonic Optical Fiber Probes

**DOI:** 10.3390/nano16100602

**Published:** 2026-05-14

**Authors:** Rosalba Pitruzzella, Dalila Cicatiello, Chiara Marzano, Federica Passeggio, Luca Gentile, José A. Ribeiro, João P. Mendes, Luís C. C. Coelho, Giuseppe Portella, Maria Chiara Capellupo, Maddalena Casale, Luigi Zeni, Pedro A. S. Jorge, Nunzio Cennamo

**Affiliations:** 1 Department of Women, Child and General and Specialized Surgery, University of Campania Luigi Vanvitelli, 80138 Naples, Italy; rosalba.pitruzzella@unicampania.it (R.P.); mariachiara.capellupo@unicampania.it (M.C.C.); maddalena.casale@unicampania.it (M.C.); 2 Department of Engineering, University of Campania Luigi Vanvitelli, Via Roma 29, 81031 Aversa, Italy; dalila.cicatiello@studenti.unicampania.it (D.C.); chiara.marzano@unicampania.it (C.M.); federica.passeggio@unicampania.it (F.P.); luigi.zeni@unicampania.it (L.Z.); 3 Department of Translational Medical Sciences, University of Naples “Federico II”, 80131 Naples, Italy; portella@unina.it; 4 DAIMEDLABTRASF, Department of Integrated Laboratory and Transfusion Medicine, Azienda Ospedaliera Universitaria Federico II, 80131 Naples, Italy; luca.gentile@unina.it; 5 Center for Applied Photonics, INESC TEC, Rua do Campo Alegre, 687, 4169-007 Porto, Portugal; jose.ribeiro@fc.up.pt (J.A.R.); joao.p.mendes@inesctec.pt (J.P.M.); luis.c.coelho@inesctec.pt (L.C.C.C.); pedro.jorge@fc.up.pt (P.A.S.J.); 6 Departamento de Física e Astronomia, Faculdade de Ciências da Universidade do Porto, Rua do Campo Alegre, 687, 4169-007 Porto, Portugal

**Keywords:** point of care test (POCT), plastic optical fiber (POF), surface plasmonic resonance (SPR), electropolymerized molecularly imprinted polymer (eMIP)

## Abstract

Haemoglobin (Hb) concentration is a key biomarker for several diseases. Traditional laboratory methods often have limitations due to their time-consuming nature, the need for skilled personnel, or the use of high-cost instrumentation. This work presents a sensing strategy for developing new point-of-care tests (POCTs) for Hb detection via a proof of concept. The proposed sensing approach is implemented using plasmonic plastic optical fiber (POF) sensor chips that integrate an electropolymerized molecularly imprinted polymer (eMIP) film on the plasmonic surface for Hb-selective detection. The developed sensor system demonstrates an ultra-low detection limit of 80 fM in buffer, about five orders of magnitude lower than that of other comparable Hb sensors. Selectivity tests against common interfering proteins, such as bovine serum albumin (BSA) and immunoglobulin G (IgG), confirmed high specificity towards the target analyte. Moreover, the sensor’s performance was tested using a whole-blood sample, yielding results consistent with those of standard haematology analysis. The proposed sensor system, based on simple equipment, provides a quick (about 10 min) and cost-effective (about 10 euros per chip) label-free diagnostic tool for POCTs in real-world scenarios, such as finger-prick sampling, offering a less invasive alternative to traditional laboratory methods, towards devices useful for Internet of Medical Things (IoMT).

## 1. Introduction

Accurate and timely quantification of haemoglobin (Hb) concentration is considered the keystone of haematological and clinical diagnostics [1]. Haemoglobin is a remarkably important protein found within red blood cells, acting as the body’s essential oxygen ferry. This tireless carrier is what enables the body to transport respiratory gases efficiently [2]. In the lungs, haemoglobin binds oxygen tightly, carrying it through the bloodstream to all tissues, where it graciously releases oxygen and, in turn, picks up carbon dioxide waste produced by cellular metabolism. This spent gas is then transported back to the lungs for exhalation and renewal [2]. Structurally, haemoglobin is a complex, compact machine, built from four protein chains (globins), each of which cradles a non-protein heme group. At the very heart of each heme lies a single iron atom, the molecular engine that critically orchestrates the binding and release of both oxygen and carbon dioxide. It is also the reason blood has a red colour [3]. The concentration of haemoglobin in the blood is an important clinical indicator of a person’s overall physiological status. When levels are too low, it may lead to anaemia, a condition that often causes fatigue, weakness, pale skin, dizziness, and difficulty breathing [4].

To aid in the diagnosis of anaemia, the World Health Organisation (WHO) establishes critical haemoglobin threshold values that serve as global clinical benchmarks. These thresholds are meticulously tailored to account for the patient’s age, sex, and physiological state. For instance, anaemia is diagnosed when haemoglobin is below 11 g/dL in children aged 6 to 59 months, below 11.5 g/dL in those aged 5 to 11 years, and below 12 g/dL in adolescents and non-pregnant women. For pregnant women, the threshold is 11 g/dL, while for men aged 15 years and older, it is 13 g/dL [2]. Adherence to these precise standards is essential for accurate clinical intervention and for globally comparable healthcare metrics. Values lower than these suggest anaemia, while unusually high levels may indicate polycythemia [3]. Anaemia remains a widespread public health issue across both low- and high-income countries [5].

According to the WHO, the causes of anaemia are multifaceted and often interrelated, including nutritional deficiencies (especially iron, vitamin B12, or folate), chronic infections and inflammation, inherited disorders of haemoglobin, blood loss, and even socioeconomic or environmental factors. Specific causes include malaria, HIV, tuberculosis, and parasitic infections, as well as demographic variables such as age, sex, and ethnicity [6]. Given this broad spectrum of etiologies, the simple yet profound act of measuring haemoglobin concentration remains the most reliable and widely used method for diagnosing anaemia, serving as a critical first line of defence in patient care. Among the many available tests, measuring haemoglobin concentration in blood remains the most reliable and widely used method for diagnosing anaemia. It is commonly performed in both hospital and outpatient settings [7]. Although the cyanomethaemoglobin method remains the gold standard due to its accuracy and standard calibration, its use of cyanide-based reagents presents safety and disposal problems. These drawbacks have consequently led to the exploration of simpler and less expensive alternatives, such as the haemoglobin colour scale, which exploits techniques based on the observation of blood colour or its transformation and compares them with well-known standard colour scales, such as the Tallqvist method [8]. Other techniques used for haemoglobin analysis involve a chemical reaction that transforms haemoglobin into a coloured compound; the intensity of these compounds can be measured or compared, for example, in Sahli’s test [8] and the Lovibond-Drabkin technique [9].

Finally, methods that exploit the correlation between blood density and haemoglobin concentration, such as the copper sulphate method, can be less precise and consistent [10]. On the other hand, modern haematology analysers offer high precision and use safer reagents such as sodium lauryl sulphate [11]. However, these systems are expensive, require regular maintenance, and need trained personnel. These aspects make them hard to access for many settings, especially in low-income or rural areas. As a result, there is growing interest in point-of-care testing (POCT) compact devices, user-friendly tools that enable immediate testing outside traditional labs [6].

In recent years, POCTs have transformed diagnostics, offering fast, reliable results directly at the patient’s location, facilitating quicker decisions and treatments, especially in emergencies or resource-limited areas [12]. In the specific field of haemoglobin measurement, POCT aims to overcome the limitations of traditional laboratory methods, such as invasive sampling and delayed results. Consequently, haemoglobin-specific POCTs have been developed, divided into invasive and non-invasive approaches [13].

Among these, invasive POCTs, such as the HemoCue, provide rapid results at the point-of-care but still require a small blood sample, usually obtained through a finger prick. A significant innovation is represented by non-invasive POCTs, such as the Masimo Pronto-7 [14]. These instruments allow haemoglobin measurement without blood sampling, using a multi-wavelength sensor placed on the finger that calculates concentration based on light absorption, similar to a pulse oximeter [15]. Beyond these direct haemoglobin measurement POCTs, the broader field of POCTs is continuously evolving, with promising new sensor technologies exploiting nanotechnologies and other innovations. One such approach for biochemical applications is the use of sensors that exploit surface plasmon resonance (SPR) phenomena combined with molecular recognition elements [16,17]. A significant innovation is the implementation of sensors exploiting modified plastic optical fibres (POFs) [17,18,19]. POFs are light, flexible, durable, and inexpensive, making them suitable for use in remote or harsh environments [18] and for easy modification to realise selective sensors [19].

The SPR-POF sensors can detect interactions at the interface of a thin plasmonic metal layer, typically gold or silver, between a chemical or biological receptor layer and the analytes of interest [19]. For example, a recent SPR-POF sensor designed to detect respiratory syncytial virus (RSV) used antibodies targeted at the virus’s F protein, demonstrating a low detection limit and high specificity, and providing results from clinical samples in about 10 min, with these results confirmed by the gold standard (RT-PCR) [20].

In addition to biological receptors, such as antibodies, chemical receptors are another category of great interest in developing POF-based POCTs [19]. In particular, molecularly imprinted polymers (MIPs), designed with specific binding sites for a specific analyte, show high chemical and physical stability, robustness under various environmental conditions, lower production costs, and longer shelf life than biological receptors (e.g., antibodies, aptamers) [21,22].

While common polymerization methods, such as thermal and photopolymerization, offer good performance in terms of sensitivity, selectivity, and stability, it has been demonstrated that experimental factors such as temperature strongly influence the quality and recognition properties of MIPs developed for protein detection [23]. Furthermore, a critical limitation for SPR sensing applications is the difficulty of precisely controlling the polymer thickness, which compromises the sensitivity, reproducibility, and reliability of sensor performance. This issue is particularly critical for SPR-based sensors, where the intensity of the evanescent field exponentially decreases with distance from the plasmonic metal surface [24], highlighting the need for highly controlled fabrication methods. In this regard, electrochemical polymerization stands out as a particularly simple, versatile and effective technique for fast depositing MIP films over metal surfaces with nanometer-scale thickness control [25], enabling large-scale production of MIP-based technologies. This process can be performed in situ via electrochemical techniques, such as chronoamperometry or cyclic voltammetry (CV), enabling the formation of a nanostructured electropolymerized MIP (eMIP) film. The film thickness can be finely tuned by adjusting the total charge passed to the electrode at a constant potential or by varying the number of CV cycles [26]. Therefore, electropolymerization provides several advantages, including precise control over polymer film thickness (essential for optimising sensor sensitivity and selectivity), versatility (allowing deposition on electrodes of various shapes and sizes), and compatibility with combined methods and rapid processes (crucial for enabling large-scale production of MIP-based technologies) [25,27].

Several examples of eMIP-based sensors for the selective recognition of several types of (bio)molecules have already been reported in the literature, including proteins [28,29,30], environmental contaminants [31], natural compounds [32], and drugs [27]. Recently, a new hybrid sensor configuration combining an eMIP and SPR-POF probe has emerged as a highly promising sensing strategy for advanced biosensing, demonstrating effectiveness in detecting analytes such as dopamine (DA) and various drugs in different matrices [33]. The advantage of SPR-POF platforms combined with eMIPs is the ability to operate without fluorescent markers or indicators, simplifying the process while maintaining high sensitivity and specificity.

In this work, the proposed SPR-POF-eMIP sensor, using DA as an electroactive monomer, was developed and tested for haemoglobin detection over a concentration range from 0.1 pM to 1000 pM. Initially, binding experiments were carried out in phosphate-buffered saline (PBS) to evaluate the sensor response using dose–response curves (calibration curves). Selectivity tests were also performed to demonstrate the eMIP selectivity properties. In particular, the proposed Hb sensor was tested, as a proof of concept, on a real sample (whole-blood), yielding results consistent with those obtained by standard haematology analysis. Finally, a comparison with state-of-the-art sensing strategies was conducted.

## 2. Materials and Methods

### 2.1. Reagents

Bovine serum albumin (BSA), dopamine (DA, 98%), haemoglobin (Hb, human) and immunoglobulin G (IgG), used as interfering substances, were purchased from Merck KGaA, Darmstadt, Germany.

Phosphate buffer solution (PBS) 0.1 mol L^−1^, pH 7.2, was prepared using sodium dihydrogen phosphate monohydrate (ACS Reagent, Merck KGaA) and sodium phosphate dibasic dihydrate (EMSURE, Supelco, Bellefonte, PA, USA) salts.

### 2.2. SPR-POF Platform Fabrication

The plasmonic probe was fabricated from a plastic optical fiber (POF) with a 980 µm polymethylmethacrylate (PMMA) core and a 10 µm fluorinated polymer cladding (total diameter 1 mm), as described in previous work [34]. To modify the POF, it was embedded in a resin block with a specific trench inside to obtain subsequent fabrication steps. In the first step, a D-shaped POF sensing region was created by polishing the cladding and a portion of the core using 5 µm and 1 µm polishing papers. Next, about a 1 µm thick layer of Microposit S1813 photoresist (MicroChem Corp., Westborough, MA, USA) was deposited by spin-coating at 6000 rpm for 60 s on the D-shaped POF region. This photoresist layer, with a higher refractive index (RI) than PMMA, was added to enhance the sensor’s plasmonic performance [34]. Finally, a 60 nm gold film was deposited by sputtering (Safematic CCU-010, Zizers, Switzerland) to obtain the SPR phenomena. The resulting optical platforms, named SPR-POF probes, have a planar sensing region that allows direct application of the pre-polymer mixture and the sample under test via a drop (about 50 μL), without the aid of a microfluidic system.

### 2.3. 3D-Printed Cell for eMIP Deposition

**Figure 1:**
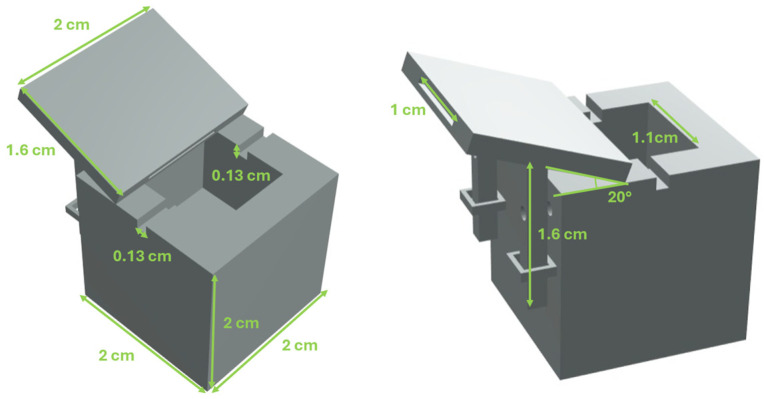
CAD image of the 3D-printed cell hosting the SPR-POF platform and the screen-printed voltammetric cell.

A custom-designed 3D-printed holder was fabricated to host the sensor and facilitate the deposition of eMIP onto its sensitive region. This custom 3D-printed holder was developed using Fusion 360 CAD software (v. 2702.1.47, Autodesk, San Francisco, CA, USA) and subsequently printed with a Photon Mono X photo-curing resin printer (Anycubic, Shenzhen, China). As shown in Figure 1, the holder design was intended to integrate the SPR-POF platform with a screen-printed voltammetric cell. This was achieved by incorporating a dedicated slot within a mechanism that precisely aligns the screen-printed cell with the SPR-POF probe’s sensitive region. The holder with the slot for the screen-printed can be removed. Figure 1 shows a CAD image with all sizes reported.

### 2.4. Experimental Setup

**Figure 2:**
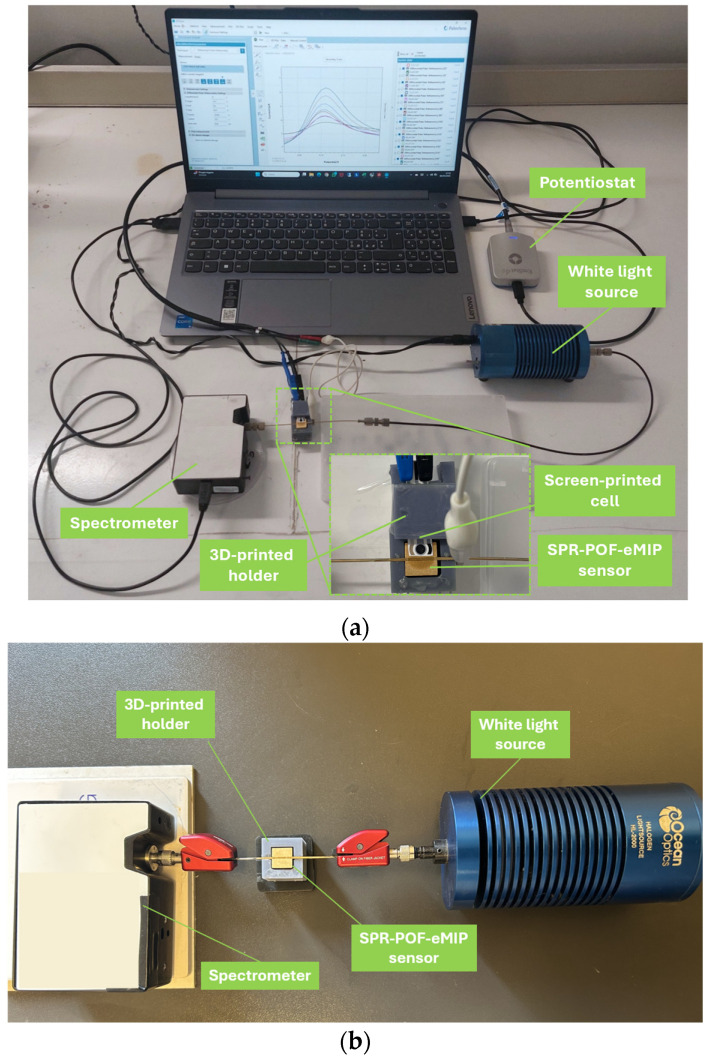
(**a**) Picture of the experimental setup used to deposit the eMIP layer over the SPR-POF platform surface and to monitor the SPR response, with a zoom inset of the SPR-POF-eMIP sensor. (**b**) Picture of the optoelectronics experimental setup used to test the SPR-POF-eMIP sensor for the haemoglobin detection in a real scenario (POCT view).

An integrated experimental setup shown in Figure 2a was used to enable the electropolymerization of the MIP layer onto the SPR-POF platform surface and to monitor the SPR response during eMIP layer formation. Screen-printed voltammetric cells (Topflight Italia S.P.A., Vidigulfo, Pavia, Italy), featuring carbon-ink counter electrodes and an Ag/AgCl-ink pseudo-reference electrode, were employed. An EmStat4s potentiostat (PalmSens BV, Houten, The Netherlands) was used for voltammetric analysis. More specifically, the SPR-POF-eMIP sensor acted as the working electrode, while the counter and pseudo-reference electrodes were integrated within the screen-printed cell. All electrodes were electrically connected via alligator clips.

Figure 2b shows the small-sized experimental setup used for optical measurements in a POCT view, for haemoglobin detection in a real scenario. The SPR-POF-eMIP sensor was housed in a 3D-printed holder, without the removable holder with the slot for the screen-printed (described in Section 2.3). It was connected, via SMA connectors, to a white light source, with an emission range of 360–1700 nm (HL2000-LL, Ocean Optics, Orlando, FL, USA), and to a spectrometer with a detection range of 350–1023 nm (SR-6VN500, Ocean Optics, Orlando, FL, USA).

### 2.5. Deposition Protocol of eMIPs and eNIPs on the SPR-POF Platforms

**Figure 3:**
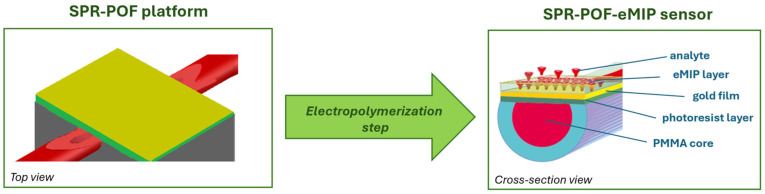
Schematic representation of the POF-based sensor before and after the eMIP deposition via the electropolymerization step. On the left, an outline of the top view of the bare SPR-POF platform with the bare gold surface. On the right, the cross-sectional view of the resulting SPR-POF-eMIP sensor chip.

Figure 3 illustrates the sensor outline before and after deposition of the eMIP layer on the SPR-POF platform surface. In particular, Figure 3 shows the top view of the bare SPR-POF platform (fabricated as described in Section 2.2) and the cross-sectional view of the SPR-POF-eMIP sensor after the MIP electropolymerization step. As shown in Figure 3, the sensing region is achieved via nanolayers deposited on the exposed cores of the POFs.

Initially, the bare gold surface of the SPR-POF platform serves as the working electrode for the electropolymerization of a MIP. In particular, 50 μL of a prepolymeric solution containing dopamine (DA) as the functional monomer and haemoglobin (Hb) molecules as the template is dropped on the gold surface. 

The MIP layer was fabricated through electropolymerization of a 2.0 mmol L^−1^ DA solution in the presence of 0.50 mg mL^−1^ Hb, prepared in 0.1 mol L^−1^ PBS, pH 7.2. The process was carried out by CV, scanning the potential from −0.7 to +0.7 V at 0.05 V s^−1^ for a total of 5 cycles.

The electropolymerization step results in the formation of a thin polymeric layer. As explained in detail in Section S1 of the Supplementary Material, the thickness characterization was performed from 5 points of ellipsometry measurements. In particular, to estimate the thickness of the electropolymerized polymer via ellipsometry, an SPR planar glass disk substrate is used. 

As described in the Supplementary Material, an average film thickness of 9.0 ± 0.5 nm was obtained with a mean squared error (MSE) of 7.5 ± 0.3, as shown in Figure S1 of the Supplementary Materials. Subsequently, the selective removal of haemoglobin through an extraction step generates specific recognition sites within the polymer matrix. 

Template removal was achieved by immersing the modified platform in 0.05 mol L^−1^ acetate buffer (pH 4.5) containing 5% (*w*/*v*) SDS, followed by overnight incubation. 

Each stage of the MIP formation was evaluated via electrochemical measurements using a 5 mmol L^−1^ hexacyanoferrate (HCF) redox probe. 

For comparison purposes, a non-imprinted polymer (NIP) film was synthesized under identical conditions, excluding the presence of the template peptide, and deposited in a similar way, in order to achieve selectivity tests.

### 2.6. Preparation of the Real Samples

The whole-blood sample was analyzed without further manipulation at the D.A.I. Medicine Laboratory (DAIMEDLAB) at the Federico II University Hospital (Naples, Italy). The result showed a haemoglobin (Hb) concentration of 12.3 g/dL, or 1.91 mM (considering a molecular weight of 64.5 kDa). Serial dilutions were made from the real sample until the detection range of the SPR-POF-eMIP sensor (pM) was reached. Specifically, the following dilutions were prepared—2 × 10^9^, 10^9^, and 2 × 10^8^—inPBS. Moreover, in this way, the dose–response curves in PBS can serve as calibration curves.

### 2.7. Measurement Protocol for Haemoglobin Determination

Haemoglobin (Hb) detection using SPR-POF-eMIP sensors was performed according to a specific measurement protocol. Initially, a reference spectrum was recorded in air, a medium in which the SPR condition was not satisfied via D-shaped POF waveguides. The reference spectrum was used to normalize the transmitted spectra acquired during the measurements. In order to obtain dose–response curves, standard haemoglobin solutions were prepared by serial dilutions in PBS, covering a concentration range from 0.1 pM to 1000 pM. For each measurement, a 50 µL aliquot of the solution was deposited onto the sensor’s sensitive area. To allow specific binding between Hb and eMIP recognition sites, the samples were incubated at room temperature for 10 min. After the incubation step, the sensor surface was washed three times with PBS to remove nonspecific interactions. The spectrum transmitted by the SPR-POF-eMIP sensor, after interaction with each Hb concentration and each relative washing step, was acquired while maintaining PBS as the bulk solution. The transmitted spectra were normalized with respect to the reference spectrum to obtain the SPR spectra and monitor the resonance wavelength shift.

For data analysis, the absolute value of the resonance wavelength shift (|Δλ|) relative to the blank solution (PBS without analyte) was determined for each Hb concentration in order to obtain the dose–response curves by plotting |Δλ| versus Hb concentration.

For the selectivity tests, the same protocol has been used for the eNIP-based sensor configuration. Moreover, to assess selectivity, the SPR-POF-eMIP sensor was tested with 10 pM concentrations of bovine serum albumin (BSA) and immunoglobulin G (IgG) in PBS, substances often found in biological fluids. Solutions of these interference substances were prepared by serial dilution in PBS, and the proposed sensor was tested using the same protocol used for the analyte, as described above.

Finally, Hb measurements in diluted real samples were addressed using the same measurement protocol used for the samples in PBS. More specifically, the real whole-blood sample has been diluted to 2 × 10^9^, 1 × 10^9^, and 2 × 10^8^ in PBS (see Section 2.6).

## 3. Results

### 3.1. Monitoring of the eMIP Deposition on SPR-POF Platforms and SEM Characterization

**Figure 4:**
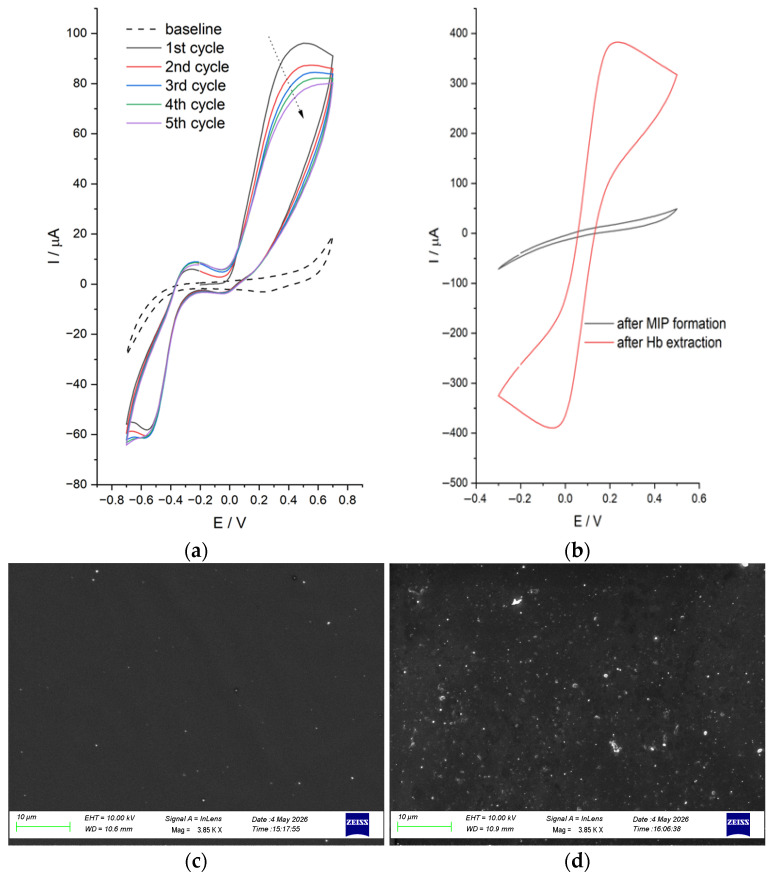
(**a**) Electropolymerization of DA solution using CV: 5 cycles. The scan rate was 50 mV s^−1^. (**b**) Representative voltammograms, obtained in the presence of 5 mmol L^−1^ HCF, after DA electropolymerization, and after template extraction. (**c**) SEM image of the bare surface of the SPR-POF probe. (**d**) SEM image of the eMIP surface of the SPR-POF-eMIP sensor.

In this work, DA was selected as an electroactive monomer to construct the eMIP film on the plasmonic chip using the surface electrochemical polymerization approach. Figure 4a shows the representative cyclic voltammograms recorded during the eMIP construction, according to the mechanisms described elsewhere [35]. 

The progressive fabrication of the eMIP imprinted layer on the plasmonic surface was validated through electrochemical characterization using CV and HCF reporting system (Figure 4b). 

The electropolymerization of dopamine enabled the straightforward formation of an eMIP layer, which initially restricted the access of the redox probe to the gold surface. Following removal of the protein, a noticeable increase in peak current was detected, indicating enhanced diffusion of the redox species through the newly created recognition cavities within the polymer matrix.

The SPR response can be used to monitor the deposition of the eMIP film. In particular, from the bare gold surface to the eMIP film deposition and extraction phases, a total resonance wavelength shift of approximately 50 nm was recorded using the same bulk solution (PBS). 

The shift in the resonance wavelength is attributable to an increase in the RI at the gold–dielectric interface, in the same bulk solution, in a similar way to [33]. 

The surface morphology of the developed sensor was studied using scanning electron microscopy (SEM), using a Zeiss Supra v35 instrument (Oberkochen, Germany). Specifically, two surfaces of sensor configurations were analyzed: the bare surface of the SPR-POF platform without a receptor (Figure 4c) and the surface of the SPR-POF-eMIP sensor (Figure 4d).

### 3.2. Dose–Response Curves of SPR-POF-eMIP Sensors

**Figure 5:**
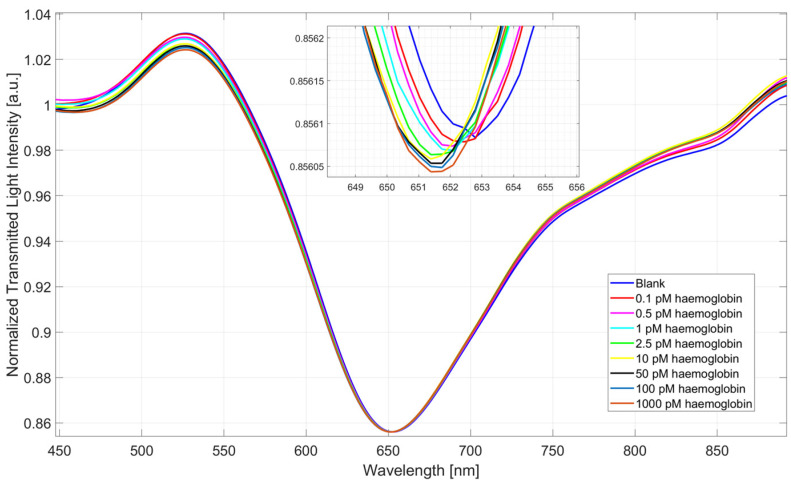
SPR spectra obtained by testing the SPR-POF-eMIP sensor with different haemoglobin concentrations in PBS, ranging from 0.1 pM to 1000 pM.

The developed SPR-POF-eMIP sensor was tested with haemoglobin concentrations in PBS ranging from 0.1 pM to 1000 pM. Figure 5 shows the SPR spectra obtained by normalizing all the transmitted spectra acquired after the incubation of different haemoglobin concentrations to a reference spectrum (the spectrum acquired in air), as described in Section 2.7.

As shown in Figure 5, the resonance wavelength exhibits a blue shift, indicating that when eMIP–analyte binding occurs, the resonance wavelength decreases. This behaviour confirms the interaction between the analyte and the imprinted recognition sites within the eMIP layer, in agreement with results for dopamine detection using another eMIP layer on the same SPR-POF platform [33].

Three different SPR-POF-eMIP sensors were fabricated using the same procedure described in Section 2.5 and tested under the same conditions, according to the protocol reported in Section 2.7, in order to evaluate the reproducibility of the proposed sensor fabrication and its binding response in terms of calibration curves.

**Figure 6:**
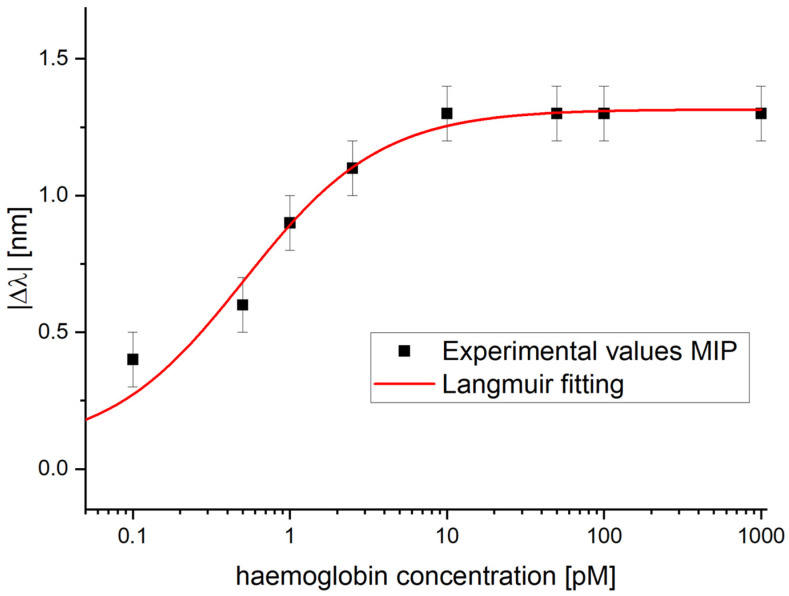
Semi-logarithmic dose–response curve in PBS for haemoglobin detection using SPR-POF-eMIP sensors. A Langmuir model fits the experimental data points.

Using these three sensor chips, Figure 6 shows the dose–response curve in PBS obtained by plotting the average absolute resonance wavelength shift (|Δλ|) relative to the blank, as a function of haemoglobin concentration.

As shown in Figure 6, the error bar was 0.1 nm and corresponds to the maximum standard deviation obtained from testing the three developed sensors (*n* = 3) under the same experimental conditions, as was done for the detection of dopamine in [33]. Batch-to-batch reproducibility was assessed by testing three sensors and comparing their responses, yielding, in the worst case, a variation in the resonance wavelength of ±0.1 nm at the same tested concentration.

This experimentally obtained error bar (the worst-case standard deviation) was not used to estimate the sensor performance parameters, but it was used to evaluate the error model reported in the fitting parameters relative to Figure 6 (see Table 1). In other words, it was useful for assessing the quality of the fitting model by comparing it with the standard deviation of the blank in the Langmuir fit, which is used to estimate the limit of detection (LOD), a key sensor chemical parameter.

The experimental values reported in Figure 6 were fitted using the Langmuir model equation with OriginPro software (OriginPro 2015 (32-bit) Srl b9.2.257, OriginLab Corp., Northampton, MA, USA), which can be defined as follows:

(1) $$\left|{\Delta \lambda }_{c}\right|=\left|\lambda_{c}-\;\lambda_{0}\right|=\left|{\Delta \lambda }_{max}\right|\;\cdot \;\frac{c}{K+c}$$

In Equation (1), λ_c_ represents the plasmonic resonance wavelength measured in the presence of a solution containing the analyte at a known concentration (*c*), while λ_0_ is the resonance wavelength measured with the blank solution (PBS without analyte). Δλ_max_ indicates the variation between the resonance wavelength at the saturation concentration of the eMIP recognition sites and that of the blank solution. *K* corresponds to the dissociation constant.

The OriginPro software automatically calculates the Langmuir fitting parameters reported in Table 1, which best match the experimental values (R^2^ = 0.98).

The Langmuir fitting values reported in Table 1 were used to estimate the three key chemical parameters of the developed Hb sensor: the affinity constant (K_aff_), the detection limit (LOD), and the sensitivity at low concentrations (S_low c_).

The K_aff_, defined by the reciprocal of K, where *K* likely represents a dissociation constant, is equal to 1.946 [pM]^−1^.

The S_low c_ was determined by the slope of Equation (1) at low concentration, when *c* is much lower than K, that is when it can be considered as a linear model. In this case, the slope of the linear equation (S_low c_ = ∣Δλ_max_∣/K) was calculated, and it is equal to 2.558 [nm/pM].

The LOD was calculated using the ratio between 3.3 times the standard error of the blank (see Table 1, considered as the error model) and the S_low c_ (LOD = $\frac{3.3\times St.error\left(\lambda_{0}\right)}{S_{low\;c}}$), and it equals 0.08 [pM]. 

These sensor chemical parameters are summarized in Table 2.

### 3.3. Selectivity Tests

To demonstrate the selectivity of the developed sensor system, based on the eMIP layer combined with the SPR-POF platform, different selectivity tests were performed.

Specifically, experimental tests were conducted using two different sensor configurations: a non-imprinted polymer configuration (SPR-POF-eNIP) and a bare gold surface configuration (SPR-POF platform without receptor). All sensor configurations (eMIP, eNIP, and bare) were tested using the same experimental setup (shown in Figure 2b), concentration range, and measurement protocol (as described in Section 2.7).

**Figure 7:**
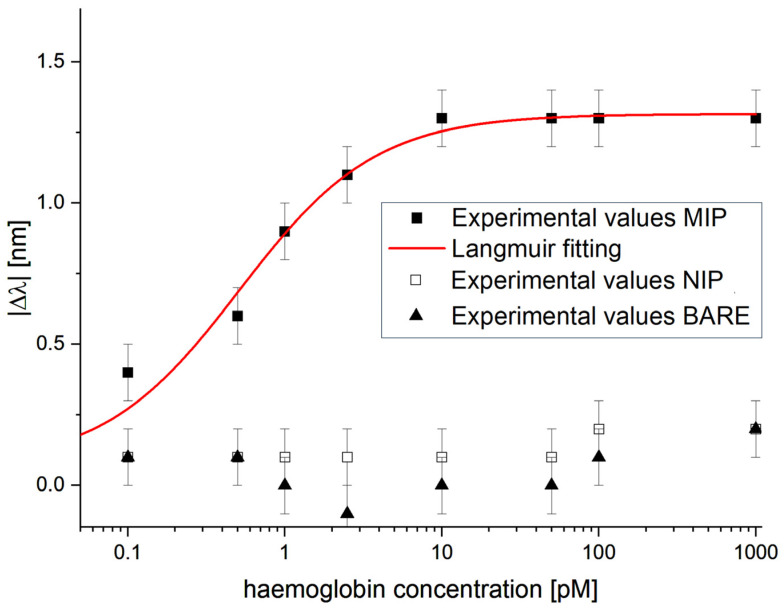
The absolute value of the resonance wavelength variations versus Hb concentration in PBS for two different sensor configurations: eNIP (open squares) and bare gold surface (black triangles). The eMIP configuration (black squares) is also shown for comparison.

Figure 7 presents the experimental values and error bars for both tested sensor configurations (eNIP and bare). For comparison, the SPR-POF-eMIP sensor’s dose–response curve is also reported.

As shown in Figure 7, no significant shift in resonance wavelength was observed in either the bare sensor or eNIP-based sensor configurations as the Hb concentration increased, indicating that the sensor’s response was specific and selective to the binding between haemoglobin and the eMIP sites.

Another selectivity test of the SPR-POF-eMIP sensor was also performed by measuring according to the protocol described in Section 2.7, using solutions containing substances other than the target analyte (Hb). Specifically, bovine serum albumin (BSA) and Immunoglobulin G (IgG) in PBS were tested at 10 pM, an order of magnitude higher than the Hb concentration value tested (1 pM) for comparison. 

**Figure 8:**
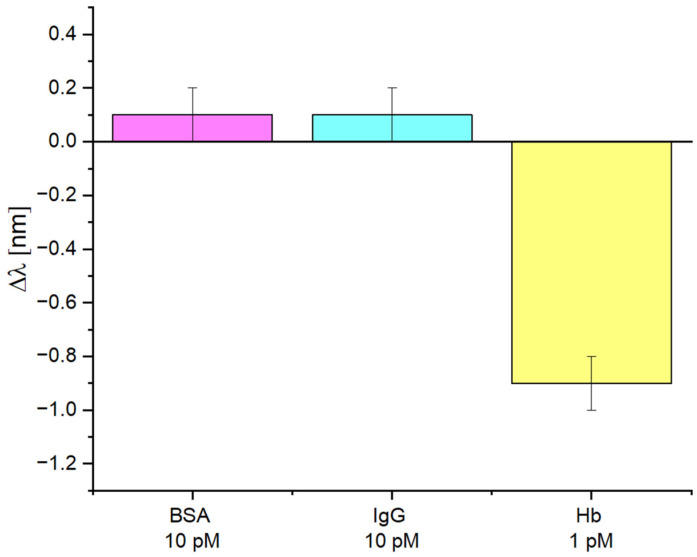
Selectivity tests in PBS: comparison between the resonance wavelength variations produced by BSA and IgG at 10 pM and those of the analyte (Hb) at 1 pM.

Figure 8 shows the experimental results in terms of resonance variations, indicating that BSA and IgG caused only a slight resonance shift, which fell within the sensor system error bar (±0.1 nm), even though they were 10 times more concentrated than the analyte solution reported in the same figure.

### 3.4. Tests on a Real Whole-Blood Sample as Proof of Concept

The binding measurements reported here were obtained from diluted real samples, specifically in a diluted whole-blood matrix. 

This proof of concept approach is crucial for evaluating its capability for future applications in a real-world POCT setting. More specifically, this test can be considered a sort of selectivity test on a complex matrix (whole-blood). 

The results obtained via the developed Hb sensor were compared with haemoglobin values determined using a standard venipuncture method, which indicated a concentration of 12.3 g/dL (1.91 mM), as reported in Table 3.

The real sample was diluted to fall within the concentration range of the developed SPR-POF-eMIP sensor and used the dose–response curve in PBS as the calibration curve, as explained in Section 2.6. 

**Figure 9:**
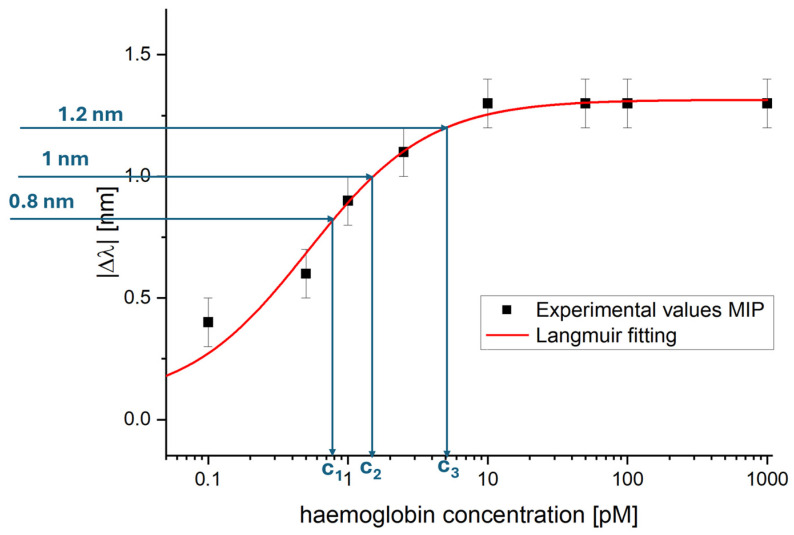
Estimation of the haemoglobin concentration in diluted whole-blood samples through the dose–response curve in PBS (as calibration curve) and the responses of the eMIP-SPR-POF sensor.

The same real sample was diluted in PBS by three dilution ratios: 2 × 10^9^, 10^9^, and 2 × 10^8^. The three diluted samples were tested according to the measurement protocol (detailed in Section 2.7), and the absolute resonance wavelength shift value relative to the blank was calculated, as reported in Figure 9 and Table 3. 

Figure 9 shows the dose–response curve obtained in PBS, which was used as a calibration curve, together with the absolute resonance shift obtained from the three diluted real samples to estimate the Hb concentration value in the real sample graphically. In particular, from the three diluted samples tested, only two fell within the sensor’s dynamic range. The first two diluted samples yielded Hb concentration estimates of 0.77 ± 0.23 pM and 1.71 ± 0.71 pM, respectively. These values, multiplied by the respective dilution factors (2 × 10^9^ and 10^9^), allowed estimating Hb concentrations in the real undiluted sample at 1.54 ± 0.46 mM and 1.71 ± 0.71 mM, respectively. The third sample analyzed fell within the saturation zone and does not allow an estimate. Table 3 summarizes the graphical procedure steps used to estimate Hb in the real whole-blood sample.

The observed consistency between the values measured by the developed SPR-POF-eMIP sensor system and those obtained through the standard method highlights the potential of the proof of principle in the analysis of small sample volumes, such as those typically obtained through capillary finger sampling, making it particularly suitable for POCT applications where speed and simplicity of sampling and analysis are key aspects.

More specifically, as shown in Table 3, only a dilution step is required to obtain the Hb concentration value.

## 4. Discussion

To evaluate the proposed SPR-POF-eMIP sensor, its analytical performance was compared with that of other state-of-the-art haemoglobin detection sensors. Table 4 summarises a comparative analysis of the different haemoglobin (Hb) detection strategies, ranging from electrochemical and amperometric sensor systems [36] to alternative SPR-based sensors [37]. In particular, the developed SPR-POF-eMIP sensor has been evaluated by comparing its LOD with those of several established methodologies, as summarised in Table 4. The proposed platform demonstrates a significant improvement in sensitivity, achieving an LOD approximately 5 orders of magnitude lower than that of most existing approaches. For example, while electrochemical techniques, such as amperometric detection using MB-MWNTs/GC electrodes [36] or cyclic voltammetry on Au/NH_2_-modified ITO [38], reported LODs in the nanomolar range (1.5 nM and 10 nM, respectively), the developed SPR-POF-eMIP sensor works at much lower concentrations. This performance gap is even more pronounced when compared with other optical platforms; for example, a previously reported SPR-based sensor [37] showed an LOD of 18.6 nM. These results suggest that integrating the eMIP film with the SPR-POF platform yields superior performance for the detection of haemoglobin at femtomolar to picomolar levels, compared to the current state-of-the-art methods.

In particular, the femtomolar range obtained in this work is similar to that achieved by exploiting the combination of nanoMIPs with the same SPR-POF platform for transferrin protein detection [38]. In other words, the thickness of the e-MIP layer is at the nanoscale, similar to nanoMIPs, thereby improving sensor performance.

The ultra-low detection limit of the developed sensor system (80 fM) can be exploited to eliminate nonspecific interactions with the sensor surface caused by real samples (whole-blood), requiring only a simple dilution step and being particularly advantageous for POCT applications.

Finally, even though this work has been developed and tested as a proof of concept, the reproducibility of the SPR-POF platform combined with the MIP layers has been extensively validated across several applications, including 2-FAL detection [19] and SARS-CoV-2 detection in the BETTER project [39]. More specifically, in the BETTER project, SARS-CoV-2 detection in Universal Transport Medium (UTM) has been confirmed using SPR-POF chips with an MIP layer specific for the SARS-CoV-2 sub1 spike protein, across about 1000 tests with disposable SPR-POF-MIP sensors, and compared with the gold standard polymerase chain reaction (PCR) method.

More specifically, regarding the eMIP layer used in this application, long-term stability, storage conditions, and shelf life are functions of the characteristics of the polymer, which is well known for its high stability.

Regarding batch-to-batch sensor reproducibility, it was verified by developing and testing several eMIPs on SPR-POF platforms, with good reproducibility (e.g., [33]). In this proof of concept, three SPR-POF-eMIP sensors (*n* = 3) were tested under identical experimental conditions, as in the detection of dopamine using a similar sensing strategy [33]. The results from testing the three sensors are completely comparable, with a worst-case variation in the resonance wavelength of ±0.1 nm at the same tested concentration.

**Table 4:** Comparison of haemoglobin sensors using different sensing strategies.

Sensor Configuration	Sensing Method Strategies	LOD	Reference
SPR-POF probes combined with eMIPs for haemoglobin	SPR	80 fM	This work
Direct detection of Hb on Au/NH_2_-modified ITO electrodes	CV	10 nM	[40]
Organic catalyst MB-MWNTs/GC modified electrodes	Amperometry (flow injection)	1.5 nM	[36]
Boronic acid-modified SPR sensor (for Hb via cis-diol binding)	SPR	18.6 nM	[37]
Miniaturized device for electrochemical impedance spectroscopy	Electrochemical impedance	206 nM	[41]

The compact system size, excellent binding sensitivity, and low cost of the developed SPR–POF-eMIP sensor system make it ideal for POCT applications in real-world settings. The main advantage of the proposed sensor is that the test can be performed with a single finger prick, reducing the need for further painful venipunctures or the use of clinical laboratories [6]. Notably, the proposed approach requires a very small volume of blood and only requires a dilution step to analyze a real sample within 10 min. This makes it easier to use than traditional procedures, such as the cyanomethaemoglobin method or automated haematology analyzers, which require expensive equipment, trained staff and infrastructure [12].

## 5. Conclusions

In this work, a sensor strategy for POCTs is demonstrated for the detection of haemoglobin at the femtomolar to picomolar level. Unlike other optical–chemical sensors based on MIPs, this work investigates the use of electropolymerization to obtain a thin eMIP film to improve the performance. The proposed sensing approach, based on a simple setup combined with disposable Hb sensor chips (SPR–POF probes combined with electropolymerized MIPs), achieves an LOD of 80 fM and good selectivity. A key point is that the analyses were also performed in a real matrix (whole-blood), demonstrating the sensor’s ability to operate effectively with small sample volumes. The experimental results, obtained as a proof of concept, demonstrate the device’s capabilities and provide a basis for future tests in real-world scenarios, such as finger-prick sampling, offering a less invasive alternative to traditional laboratory methods.

The proofofprinciple is useful to develop POCTs for biomedical applications. Moreover, the developed sensor system could involve a wider population by connecting the device to the Internet to provide POCT, which is useful for the Internet of Medical Things (IoMT).

## Figures and Tables

**Table 1:** The Langmuir fitting parameters relative to Hb detection by the SPR-POF-eMIP sensor.

λ_0_ [nm]		Δλ_max_ [nm]		K [pM]		Statics	
Value	St.error	Value	St.error	Value	St.error	χ^2^	R^2^
0.069	0.062	1.315	0.037	0.514	0.108	0.13	0.98

**Table 2:** Chemical parameters of the developed SPR-POF-eMIP sensor.

Chemical Parameters	Value
K_aff_	1.946 [pM]^−1^
S_low c_	2.558 [nm/pM]
LOD	0.080 [pM] (80 fM)

**Table 3:** Summary of the SPR-POF-eMIP responses to a whole-blood sample at different dilution factors, together with the estimated Hb concentration via the sensor calibration curve.

Diluted Sample	|Δλ| [nm]	Estimated Hb Concentration of the Diluted Sample [pM]	Dilution Factor	Estimated Hb Concentration of the Real Sample [mM]	Concentration Value of the Sample via the Gold Standard
Whole-blood sample diluted 1:2 × 10^9^	0.8 ± 0.1	c_1_ = 0.77 ± 0.23	2 × 10^9^	1.54 ± 0.46	12.3 g/dL (1.91 mM)
Whole-blood sample diluted 1:10^9^	1 ± 0.1	c_2_ = 1.71 ± 0.71	10^9^	1.71 ± 0.71	
Whole-blood sample diluted 1:2 × 10^8^	1.2 ± 0.1	c_3_ = near the saturation value	2 × 10^8^	-	

## Data Availability

The original contributions presented in this study are included in the article/Supplementary Material. Further inquiries can be directed to the corresponding author.

## Supplementary Materials

The following supporting information can be downloaded at: https://www.mdpi.com/article/10.3390/nano16100602/s1, Section S1: Polymer thickness estimation; Figure S1: Ellipsometry image of the thickness vs. position obtained for the polydopamine NIP film electropolymerized on the gold SPR substrates.
