# Supplementary material for: Towards Haemoglobin Detection in Finger-Prick Sampling via Low-Cost Disposable Sensor Chips Based on eMIPs on Plasmonic Optical Fiber Probes
Source: Nanomaterials (Basel). 2026 May 14;16(10):602. doi: 10.3390/nano16100602 (PMC13210363; doi:10.3390/nano16100602)
Supplement: Supplementary file 1 [file nanomaterials-16-00602-s001.zip › nanomaterials-4280127-supplementary.pdf]

nanomaterials

MDPI

Supplementary materials

Towards Haemoglobin Detection in Finger-Prick Sampling via Low-Cost Disposable Sensor Chips Based on eMIPs on Plasmonic Optical Fiber Probes

Rosalba Pitruzzella[1,2], Dalila Cicatiello[2], Chiara Marzano[2], Federica Passeggio[2,3], Luca Gentile[4], José A. Ribeiro[5,6], João P. Mendes[5], Luís C. C. Coelho[5,6], Giuseppe Portella[3], Maria Chiara Capellupo[1], Maddalena Casale[1], Luigi Zeni[2], Pedro A.S. Jorge[5,6], Nunzio Cennamo[2,*]

[1] Department of Women, Child and General and Specialized Surgery, University of Campania Luigi Vanvitelli, 80138 Naples, Italy; rosalba.pitruzzella@unicampania.it (R.P.); mariachiara.capellupo@unicampania.it (M.C.C.); maddalena.casale@unicampania.it (M.C.)
[2] Department of Engineering, University of Campania Luigi Vanvitelli, Via Roma 29, 81031 Aversa, Italy; dalila.cicatiello@studenti.unicampania.it (D.C.); chiara.marzano@unicampania.it (C.M.); federica.passeggio@unicampania.it (F.P.); luigi.zeni@unicampania.it (L.Z.)
[3] Department of Translational Medical Sciences, University of Naples "Federico II", 80131 Naples, Italy; portella@unina.it
[4] DAIMEDLABTRASF, Department of Integrated Laboratory and Transfusion Medicine, Azienda Ospedaliera Universitaria Federico II, 80131 Naples, Italy; luca.gentile@unina.it
[5] Center for Applied Photonics, INESC TEC, Rua do Campo Alegre, 687, 4169-007 Porto, Portugal; jose.ribeiro@fc.up.pt (J.A.R.); joao.p.mendes@inesctec.pt (J.P.M.); luis.c.coelho@inesctec.pt (L.C.C.C.); pedro.jorge@fc.up.pt (P.A.S.J.)
[6] Departamento de Física e Astronomia, Faculdade de Ciências da Universidade do Porto, Rua do Campo Alegre, 687, 4169-007 Porto, Portugal

* Correspondence: nunzio.cennamo@unicampania.it

Academic Editor: Alexander B. Kotlyar

Received: 9 April 2026
Revised: 4 May 2026
Accepted: 9 May 2026
Published: 14 May 2026

Copyright: © 2026 by the author. Licensee MDPI, Basel, Switzerland. This article is an open access article distributed under the terms and conditions of the Creative Commons Attribution (CC BY) license.

1. Polymer thickness estimation

In this work, the thickness of the polydopamine film was estimated using ellipsometry. Surface plasmon resonance (SPR) gold substrates (diameter: 24 mm, KEI bv, the Netherlands) were used for ellipsometry measurements, with the same optimised electropolymerization conditions (monomer concentration, CV scan rate, number of CV cycles, electrolyte, etc.) maintained. To electropolymerize the NIP polymer film on the AuSPR chips, a conventional 3-electrode electrochemical cell was used. A saturated calomel electrode (SCE) was used as the reference electrode, a gold wire as the counter electrode, and flat SPR glass disks coated with a thin gold film (≈50 nm) as the working electrode. Prior to the electrochemical experiments, the AuSPR substrates were washed thoroughly with pure water and ethanol, then dried under N2 flow.

Ellipsometry studies were performed through an M-2000-V rotating compensator Spectroscopic Ellipsometer (J. A. Woollam, Co., Inc.), equipped with WVASE software (J. A. Woollam, Co., Inc.) for control and data analysis. Measurements were made in the wavelength range of 370 – 1000 nm at three different angles of incidence (45º, 50º and 55º). To estimate film thickness, we used a two-layer substrate-film optical model, with the film layer data fitted to a B-Spline model, as it is particularly effective for partially transparent and absorbing

organic films, such as polydopamine. The average thickness was estimated from 5 points of ellipsometry measurements (Figure S1). An average film thickness of 9.0 ± 0.5 nm was obtained with a mean squared error (MSE) of 7.5 ± 0.3.

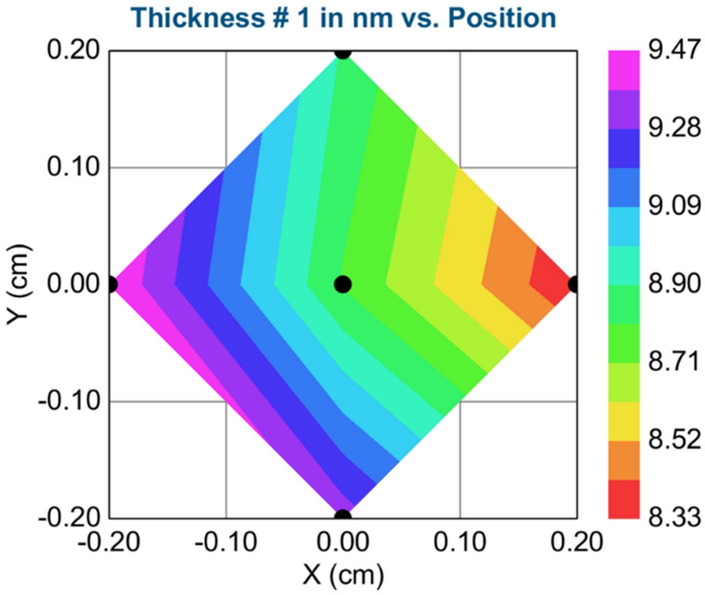

Figure S1. Ellipsometry image of the thickness vs. position obtained for the polydopamine NIP film electropolymerized on the gold SPR substrates.
